# Knowledge, Attitudes, and Practices About COVID-19 Among Healthcare Workers in Iran During the First Wave of the Pandemic

**DOI:** 10.3389/fpubh.2022.827817

**Published:** 2022-03-16

**Authors:** Hossein Hatami, Ali-Asghar Kolahi, Seyyed-Hadi Ghamari, Mohsen Abbasi-Kangevari

**Affiliations:** ^1^Department of Public Health, School of Public Health, Shahid Beheshti University of Medical Sciences, Tehran, Iran; ^2^Social Determinants of Health Research Center, Shahid Beheshti University of Medical Sciences, Tehran, Iran

**Keywords:** COVID-19, healthcare workers, knowledge, practice, SARS-CoV-2

## Abstract

**Background and Objective:**

Investigating the knowledge, attitudes, and practices about the coronavirus disease 2019 (COVID-19) among healthcare workers (HCWs) could be an early step toward identifying their potential educational needs and possible factors involved in misinformation. The objective of this study was to investigate the knowledge, attitudes, and practices about COVID-19 among healthcare workers in Iran during the first wave of the pandemic.

**Materials and Methods:**

The current descriptive-correlational study was conducted during the 1st days of the COVID-19 epidemic in Iran from March 24th to April 3rd, 2020. Participants included all healthcare workers at hospitals, including physicians, dentists, pharmacists, nurses, midwives, laboratory and radiology assistants, and other hospital professionals during the study period. Data were collected through an online self-administrative questionnaire.

**Results:**

The responses of 1,310 participants were analyzed, of which 900 (68.7%) were female. The mean (SD) knowledge score was 25.4 (3.3), 84.7% out of 30. More than 90% of participants correctly recognized the main symptoms, transmission route, and preventive measures for COVID-19. The mean (SD) attitude score was 16.9 (1.1), 93.9% out of 18. Most participants agreed with keeping safe physical distancing, self-isolation upon symptom onset, and city lockdowns. The mean (SD) score for general practices about COVID-19 was 20.8 (2.0), 86.7% of 24.

**Conclusion:**

The knowledge and practice of HCWs were appropriate, and their attitudes were mainly positive. However, there is still room for improvement regarding concerning misinformation and quackeries about COVID-19.

## Introduction

The coronavirus disease 2019 (COVID-19) rapidly turned into a pandemic with catastrophic aftermath (1), resulting in 359 M confirmed cases and 5.62 M deaths worldwide (1). To contain the community-level spread of SARS-COV-2 and alleviate the overburdening of health care systems, physical distancing measures along with school closures and lockdown have been implemented in many countries (2).

Since the beginning of the crisis, healthcare workers (HCWs) have been the front-line defense in treating patients with COVID-19 to help mitigate and control the infection (3). The battle against COVID-19 has resulted in an increased risk of infection, along with fear of SARS-CoV-2 transmission to family members of HCWs (4). The risk of a positive test for COVID-19 was increased among front-line HCWs; thus, all the necessary measures must be taken to minimize infection spread among HCWs (5).

The transmission risk of COVID-19 among HCWs is positively associated with overcrowding, inadequate ventilation facilities, and environmental contamination. Nevertheless, this risk is likely fueled by insufficient knowledge about COVID-19, especially the infection prevention practices. HCWs' knowledge about COVID-19 could also affect their attitudes and practices. Improper attitudes and practices could directly or indirectly put HCWs at risk of infection (6). Furthermore, HCWs are considered valuable sources of health education for the public. Thus, their knowledge, attitude and practice related to COVID-19 could also indirectly impact the healthcare authorities' response to COVID-19 (7).

Investigating the knowledge, attitudes, and practices about COVID-19 among HCWs could be an early step toward identifying their potential educational needs and possible factors involved in misinformation, stigmatization, and improper practices (8, 9). The objective of this study was to investigate the knowledge, attitudes, and practices about COVID-19 among healthcare workers in Iran in the early days of the pandemic via an online survey.

## Materials and Methods

This study was approved by the Ethical Committee of Shahid Beheshti University of Medical Sciences under code IR.SBMU.RETECH.REC.1399.1258. Participation was anonymous and upon the participant's own decision.

### Setting and Sampling

This anonymous network-sampling survey was carried out in the early days of the COVID-19 pandemic from March 24th to April 3rd, 2020, in Iran. Data collection was conducted via an online self-administrative questionnaire in the Google Docs platform. An online invitation post including a link to the questionnaire was circulated on the groups with healthcare workers on popular social networks in Iran, including Telegram, WhatsApp, Instagram, Twitter, and LinkedIn. To initiate the circulation, a network of medical students from all 31 provinces of Iran were asked to send the invitation to healthcare workers. Participants were selected via convenience sampling method and included healthcare workers with professional doctorate degrees, including physicians, dentists, and pharmacists; and healthcare workers without professional doctorate degrees, including nurses, midwives, laboratory and radiology assistants, and other professionals working at hospitals who were of Iranian nationality, were currently working in Iran, and agreed to participate in the study. From the Cochran formula, a 5% type I error was considered and p and q were set at 0.5, and the total sample size was calculated to be 1,440.

### Variables and the Questionnaire

Variables included sociodemographic characteristics, knowledge about COVID-19, attitudes toward COVID-19, and practices during the pandemic. Sociodemographic characteristics included participants' age, sex, ethnicity, education, and marital status.

The knowledge section included questions about symptoms of COVID-19, signs, factors associated with poor prognosis, their self-evaluation about their knowledge, source of information and statements on general knowledge.

The attitudes section enclosed statements about attitudes toward COVID-19. In response to these statements, participants could choose an item from the five-point Likert scale. The section included questions regarding their attitude toward required preventive measures including social distancing, handwashing, city lockdown, closure of schools, businesses and universities, online shopping, family visits and travel during the pandemic, and their attitudes toward source of the pandemic, religious beliefs, perspective about the pandemic future and feeling ashamed by getting sick during pandemic.

The practices section included questions about self-isolation and care-seeking, preventive measures, wearing facemasks, handwashing, leaving town, and general practices about COVID-19 (8, 9).

A panel of experts evaluated the content validity of the questionnaire. An item discrimination analysis was conducted for each scale to eliminate too tricky or easy items. Factor analysis was performed for factor structure. Separate test-retest over 2 weeks were held for the three scales of the questionnaire. The test-retest correlation for the knowledge scale was 0.86; Kuder-Richardson-20 was used to prevent the overestimation of internal consistency; the coefficient was 0.85. The test-retest correlation for the attitudes scale was 0.87; the coefficient alpha was 0.89. The test-retest correlation for the practice scale was 0.91; the coefficient alpha was 0.92. The pilot survey was conducted on 20 men and 20 women, who were recruited via convenience sampling method. The responses of the pilot population were not included in the final study.

### Data Analysis

To score the knowledge about COVID-19, one point was awarded to each correct answer. To analyze the attitudes, “I strongly agree” and “I agree” were considered as “I agree”; and “I strongly disagree” and “I disagree” were considered as “I disagree” and one point was awarded to each correct answer. To score the practices about COVID-19, one point was awarded to taking each right measure or not taking each wrong measure. For the key proportions using the exact binomial distribution, the 95% confidence interval (95% CI) was reported. Categorical variables were analyzed by the Chi-Square test. For analyzing the differences among means of two groups and three groups or more, an independent-sample *t-*test and one-way analysis of variance (ANOVA) test was used, respectively. Statistical analyses were performed using IBM SPSS Statistics version 21.

## Results

From 1,440 participants, 76 were excluded from the study due to not completing the questionnaire, and 54 due to residence in countries other than Iran at the time of the study. Thus, the responses of 1,310 participants were analyzed, among whom 900 (68.7%) were female. The mean (SD) age of participants was 29.4 (10.4): being 30.4 (10.3) among men and 27.3 (10.0) among women. Other sociodemographic characteristics of participants, including marital status, ethnicity, education, and gender, are presented in Table 1.

**Table 1:** Sociodemographic characteristics of participants.

**Variable**	**N (%)**
**Sex**	
Female	900 (68.7)
Male	410 (31.3)
**Marital status**	
Never married	804 (61.4)
Engaged	30 (2.3)
Married	451 (34.4)
Divorced	19 (1.5)
Widowed	6 (0.4)
**Ethnicity**	
Fars	817 (62.4)
Turk/Azari	221 (16.9)
Lor	74 (5.6)
Kurd	72 (5.5)
Other	126 (9.6)
**Education**	
Midwifery	87 (6.6)
Nursery	168 (12.8)
Medical Doctorate	416 (31.8)
Pharmacology and dentistry	160 (12.2)
Radiology and laboratory sciences	190 (14.5)
Ph.D. and higher	52 (4.0)
Other	239 (18.1)

### Knowledge

#### Symptoms of COVID-19

More than 95% of all HCWs have known the prevalent symptoms of COVID-19, including fever (97.1%), cough (95.2%), and dyspnea (95.8%); however, <50% of participants recognized the rare symptoms of COVID-19: sneeze, rhinorrhea, loss of consciousness and confusion. Although 93% of HCWs reported the worsening of dyspnea as a red flag of COVID-19, <80% of participants recognized that severe COVID-19 could present itself by a persistent fever, exacerbation of coughs, and productive coughs (Table 2).

**Table 2:** Knowledge of participants regarding famous symptoms and red flags of COVID-19.

**Symptoms/Red flags**	**Number of respondents**	**Yes (%)**	**No (%)**
**Symptoms**			
Fever	1,304	1,272 (97.5)	32 (2.5)
Dyspnea	1,304	1,255 (96.2)	49 (3.8)
Cough	1,304	1,247 (95.6)	57 (4.4)
Loss of smell or taste	1,304	981 (75.2)	323 (24.8)
Myalgia	1,304	846 (64.9)	458 (35.1)
Malaise	1,304	917 (70.3)	387 (29.7)
Sore throat	1,304	818 (62.7)	486 (37.3)
Diarrhea	1,304	684 (52.5)	620 (47.5)
Loss of appetite	1,304	658 (50.5)	646 (49.5)
Sneeze	1,304	304 (23.3)	1,000 (76.7)
Rhinorrhea	1304	302 (23.2)	1002 (76.8)
Loss of consciousness	1,304	390 (29.9)	914 (70.1)
Confusion	1,304	334 (25.6)	970 (74.4)
**Symptoms of severe disease**			
Exacerbating dyspnea	1,301	1,293 (99.4)	8 (0.6)
Exacerbating coughs	1,301	908 (69.8)	393 (30.2)
Persistent fever	1,301	928 (71.3)	373 (28.7)
Loss of consciousness	1,301	513 (39.4)	788 (60.6)
Confusion	1,301	199 (15.3)	1,102 (84.7)
Productive coughs	1,301	80 (6.1)	1,221 (93.9)

#### Susceptibility to Severe COVID-19

Almost 80% of HCWs recognized the factors of making patients susceptible to severe COVID-19, including ages of more than 60, diabetes mellitus, and chemotherapy. However, only 851 (65.6%) of HCWs reported hypertension as a susceptible factor. In addition, obesity and overweight were recognized as a predisposing factor by 527 (40.6%) participants (Table 3).

**Table 3:** Knowledge of participants regarding factors making patients susceptible to COVID-19.

**Factors**	**Number of respondents**	**Yes (%)**	**No (%)**
Respiratory disease	1,297	1,215 (93.7)	82 (6.3)
Age more than 60	1,297	1,199 (92.4)	98 (7.6)
Diabetes mellitus	1,297	1,126 (86.8)	171 (13.2)
Cardiovascular disease	1,297	1,069 (82.4)	228 (17.6)
Chemotherapy	1,297	1,103 (85.0)	194 (15.0)
Corticosteroids treatment	1,297	1,089 (84.0)	208 (16.0)
Transplant	1,297	1,063 (82.0)	234 (18.0)
High blood pressure	1,297	851 (65.6)	446 (34.4)
Severe obesity	1,297	527 (40.6)	770 (59.4)

#### Estimations on Pathogenicity, Hospitalization Rate, Case Fatality, and Production Number

Table 4 provides the responses of HCWs regarding the estimation of pathogenicity, hospitalization rate, case fatality, and production number of COVID-19.

**Table 4:** Participant's estimation on pathogenicity, hospitalization rate, case fatality, and production number.

**Estimates**	**Mean (SD)**	**Median (IQR)**
Pathogenicity of COVID-19 (%)	55.3 (30.5)	60.0 (20–80)
Hospitalization rate of COVID-19 (%)	25.9 (20.6)	20.0 (10–30)
Case-fatality rate of COVID-19 (%)	10.4 (14.6)	5.0 (2.0–10.0)
Production number of COVID-19 (n)	55.9 (280.0)	10.0 (4–20)

#### High-Risk Settings for COVID-19 Transmission

Almost all participants (>90%) have recognized the vulnerable places regarding COVID-19 transmission, including hospitals, sports complexes, religious facilities, and public transportation; however, only 915 (69.8%) of HCWs have reported schools as a high-risk setting.

#### Source of Information

When the participants were asked about their main routes and sources of information about COVID-19, 636 (48.5%) said Telegram, 578 (44.1%) audiovisual media including radio and television, 374 (28.5%) Instagram, 297 (22.7%) scientific articles, 293 (22.4%) news agencies, 141 (10.8%) pamphlets and posters, 98 (7.5%) print media and 74 (5.6%) Twitter.

#### General Knowledge About COVID-19

More than 90% of participants correctly responded to the questions regarding the cause of COVID-19, its route of transmission, and major preventive care. However, some 113 (8.6%) participants said eating garlic would prevent them from getting COVID-19. The mean (SD) knowledge score was 25.4 (3.3), range = 4–30, 84.7% out of 30. Considering the question about their self-evaluation on the level of knowledge about COVID-19, 422 (32.2%) considered their knowledge was sufficient, 770 (58.8%) fair, and 118 (9.0%) insufficient. The mean (SD) knowledge score of participants with professional doctorate degrees was significantly higher than other educational levels [26.3 (2.7); 95% CI: 26.1–26.5 vs. 24.6 (3.4); 95% CI: 24.4–24.9, *p* < 0.001]. In addition, the mean (SD) knowledge score of participates who declared their knowledge about COVID-19 sufficient, was higher [26.4 (2.7); 95% CI: 26.1–26.6 vs. 24.9 (3.4); 95% CI: 24.7–25.1, *p* < 0.001]. No significant differences were observed between the knowledge scores of students and their other sociodemographic variables (Table 5).

**Table 5:** Frequency of participants' answers to questions about general knowledge about COVID-19.

**Statement**	**True (%)**	**False (%)**	**Don't know (%)**
The major transmission route of COVID-19 cause is via respiratory droplets and contacts with infected	1,304 (99.5)	4 (0.3)	2 (0.2)
All patients with COVID-19 and who have been in their close contacts need to be isolated for at least 14 days.	1,291 (98.5)	5 (0.4)	14 (1.1)
Infected travelers could transmit COVID-19 cause to other healthy companions.	1,290 (98.5)	11 (0.8)	9 (0.7)
The main cause of COVID-19 is virus.	1,306 (99.7)	1 (0.1)	3 (0.2)
No medications have been approved for the prevention of COVID-19 yet.	1,231 (94.0)	23 (1.8)	56 (4.2)
No medications have been approved for the treatment of COVID-19 yet.	1,250 (95.4)	17 (1.3)	43 (3.3)
Solely washing hands with water is not enough for sanitization.	1,076 (82.1)	213 (16.3)	21 (1.6)
Using the hand dryer would not remove the pathogen.	1,078 (82.3)	159 (12.1)	73 (5.6)
Vaccination against pneumonia or influenza does not affect the protection against COVID-19.	1,116 (85.2)	36 (2.7)	158 (12.1)
Eating garlic does not prevent people from the pathogen.	1,049 (80.1)	113 (8.6)	148 (11.3)
Insects is not a proper transmission route of COVID-19 cause.	844 (64.4)	125 (9.5)	341 (26.1)
The Coronaviridae family could harm both humans and animals and cause zoonotic diseases.	797 (60.8)	324 (24.7)	189 (14.5)
Nasal wash with a saline solution would prevent people from COVID-19.	512 (39.1)	594 (45.3)	204 (15.6)
Using mouthwash has a protective effect against COVID-19	398 (30.4)	673 (51.4)	239 (18.2)
This form of COVID-19 cause has been long existing in the environment and causing infections among humans.	332 (25.3)	852 (65.1)	126 (9.6)
COVID-19 is always mild.	115 (8.8)	1,118 (85.3)	77 (5.9)
Alcohol-based solutions are more potent than soap for hand sanitizing.	155 (11.8)	1,077 (82.2)	78 (6.0)
COVID-19 in all affected patients has a severity comparable to that of the common cold.	81 (6.2)	1,207 (92.1)	22 (1.7)
Once getting COVID-19 make patients immune.	82 (6.3)	1,002 (76.4)	226 (17.3)
Patients with COVID-19 would not spread disease unless they progress fever.	87 (6.6)	1,188 (90.7)	35 (2.7)
All patients with COVID-19 will admitted at hospitals.	37 (2.8)	1,259 (96.1)	14 (1.1)
A hot shower could eliminate the existing COVID-19 causes inside the body.	88 (6.7)	1,132 (86.4)	90 (6.9)
A person with no cough, fever, or dyspnea would not have the pathogen or carry it.	34 (2.6)	1,264 (96.5)	12 (0.9)
COVID-19 would not affect the children.	65 (5.0)	1,208 (92.2)	37 (2.8)
A steam room or sauna could eliminate the existing COVID-19 causes inside the body.	55 (4.2)	1,162 (88.7)	93 (7.1)
Warming upper airways with a blow dryer could eliminate the existing COVID-19 causes inside the body.	38 (2.9)	1,212 (92.5)	60 (4.6)
Drinking alcoholic beverages could eliminate the existing COVID-19 causes inside the body.	12 (0.9)	1,257 (96.0)	41 (3.1)
It is not required for a person recovered from COVID-19 to follow the preventive guidelines.	2 (0.2)	1,278 (97.5)	30 (2.3)

### Attitudes

When participants were asked about their reaction if they would get COVID-19, 1,213 (92.6%) considered self-isolation and resting at home, 1,148 (87.6%) seeing a doctor in case symptoms get worse, and 30 (2.3%) continuing daily life.

Considering the question asking the possibility of bioterrorism purposes of COVID-19, 238 (18.2%) said very high, 283 (21.5%) high, 357 (27.3%) fair, 192 (14.7%) low, and 240 (18.3%) very low.

In response to the question considering the danger level of the current pandemic, 552 (42.1%) said very high, 611 (46.7%) high, 139 (10.6%) fair, 7 (0.5%) low, and 1 (0.1%) very low.

As many as 1,170 (89.3%) participants reported that nations would finally defeat the disease. Regarding the period of time it would take to control, 3 (0.2%) said <1 months, 277 (21.1%) 1–3 months, 490 (37.5%) 3–6 months, 176 (13.4%) 6–9 months, 148 (11.3%) 9–12 months, 190 (14.6%) >1 year, and 25 (1.9%) had no idea.

Most respondents had positive attitudes toward COVID-19. The mean (SD) score for attitudes about COVID-19 was 16.9 (1.1), range = 13–18, which is 93.9% of 18 (Table 6). The majority of participants agreed with keeping safe physical distancing, self-isolation upon symptom onset, and city lockdowns (Table 6).

**Table 6:** Attitudes of health care workers regarding the COVID-19 pandemic.

**Statement**	**Number of respondents**	**I agree (%)**	**I disagree (%)**
Physical distancing is required for all citizens.	1,306	1,303 (99.8)	3 (0.2)
Taking preventive measures is just essential to people who are at risk of severe COVID-19.	1,305	23 (1.8)	1,282 (98.2)
As soon as the appearance of COVID-19 symptoms, I would notify my employer.	1,282	1,279 (99.8)	3 (0.2)
I would pay my friend or family with COVID-19 a visit.	1,282	2 (0.2)	1,280 (99.8)
It is essential that every household member dry hands and face using a single-use towel or facial tissue.	1,278	1,269 (99.3)	9 (0.7)
I would allow my employees to leave their work as soon as their COVID-19 symptoms appear.	1,285	1,280 (99.6)	5 (0.4)
The shutdown of businesses, schools, colleges, and universities is the slightest opportunity to meet with family and friends.	1,274	69 (5.4)	1,205 (94.6)
Relatives of a person who passed away from COVID-19 should not feel ashamed.	1,267	1,251 (98.7)	16 (1.3)
Immediate lockdown of all affected cities is a necessity.	1,215	1,172 (96.5)	43 (3.5)
Other more vexing issues like road traffic collision or non-communicable diseases epidemic should be of paramount importance to authorities than the COVID-19 epidemic.	1,221	1,181 (96.7)	40 (3.3)
Taking special care of pets' contact with people is a duty of pet owners.	1,158	1,099 (94.9)	59 (5.1)
Contacts of people with confirmed coronavirus or traveling history of affected regions should be reported to healthcare authorities.	1,150	1,077 (93.7)	73 (6.3)
Personally speaking, online shopping is preferred than shopping-in-person.	1,138	1,091 (95.9)	47 (4.1)
I would inform healthcare authorities if my employer forcibly demands the illegal reopening of business.	1,080	1,050 (97.2)	30 (2.8)
I would use a homemade facemask if other types went on shortage.	1,068	901 (84.4)	167 (15.6)
Although I keep my distance, I do not feel comfortable contacting patients recovered from COVID-19.	1,054	543 (51.5)	511 (48.5)
This pandemic is a plague for humankinds' sins.	982	92 (9.4)	890 (90.6)
The pandemic would be contained by its own as the weather goes warming.	859	57 (6.6)	802 (93.4)

### Practice

#### Preventive Measures for COVID-19

Table 7 provides the responses regarding preventive measures for COVID-19 taken by HCWs. Among those who reported wearing facemask as a preventive measure, 236 (66.1%) reported wearing surgical masks, 114 (31.9%) N95 masks, 81 (22.7%) cloth masks, and 35 (9.8%) homemade masks; however, 25 (7.0%) said wearing no masks. The mean (SD) duration of waring each mask was 4.8 (5.0) hours, median (IQR) = 3.0 (2.0–6.0).

**Table 7:** Preventive measures of healthcare workers for COVID-19.

**Factors**	**Number of respondents**	**Yes (%)**	**No (%)**
Staying at home	1,310	1,261 (96.3)	49 (3.7)
Practicing hand hygiene	1,310	1,260 (96.2)	50 (3.8)
Keeping safe physical distance	1,310	1,035 (79.0)	275 (21.0)
Wearing gloves	1,310	1,009 (77.0)	301 (23.0)
Wearing a facemask	1310	1,000 (76.3)	310 (23.7)
Taking vitamin supplements	1,310	627 (47.9)	683 (52.1)
Drinking herbal tea	1,310	264 (20.2)	1,064 (79.8)
Taking Imam-Kazem-drug	1,310	13 (1.0)	1,297 (99.0)
Performing wet cupping	1,310	4 (0.3)	1,306 (99.7)
Taking violet oil	1,310	3 (0.2)	1,307 (99.8)

Among responders, the mean (SD) and median (IQR) times of daily handwashing were 13.1 (12.9) and 10.0 (6.0–15.0), respectively. In addition, participants reported the mean (SD) duration of handwashing was 23.3 (12.3), median (IQR) = 20.0 (20.0–30.0).

The mean (SD) times of daily hand-rub with alcohol-based hand sanitizers among participants was 5.7 (11.2), median (IQR) = 3.0 (1.0–6.0), respectively. The mean (SD) duration of hand-rub was 12.2 (11.4) seconds, median (IQR) = 10.0 (5.0–20.0), respectively.

#### General Practices About COVID-19

The mean (SD) score for general practices about COVID-19 was 20.8 (2.0), range = 10–24, which is 86.7% of 24 (Table 8). The mean (SD) practice score of participates whose source of knowledge was scientific articles was higher 23.4 (2.3); 95% CI: 23.1–23.6 vs. 21.9 (1.8); 95% CI: 21.7–22.1, *p* < 0.001. No significant differences were observed between practice scores of participants and their other socioeconomic features.

**Table 8:** General statements regarding practices of participants about COVID-19.

**Statement**	**Yes (%)**	**No (%)**
I will not travel during Nowruz.	1,273 (97.2)	37 (2.8)
I will quarantine myself at home if I progress a symptom of COVID-19.	1,262 (96.3)	48 (3.7)
I cover my mouth and nose when sneezing or coughing.	1,247 (95.2)	63 (4.8)
I sanitize the packaging of everything I buy from stores.	84 (6.4)	1,226 (93.6)
I did not travel during Nowruz.	1,232 (94.0)	78 (6.0)
I always follow the handwashing protocols before touching my face.	1,181 (90.2)	129 (9.8)
I stayed at home as soon as I was asked by authorities.	1,173 (88.5)	137 (11.5)
I regularly sanitize my cellphone according to guidelines.	1,115 (85.1)	195 (14.9)
I carry hand-sanitizers or water-soap solutions when I go outside.	1,078 (82.3)	232 (17.7)
I always use a facemask leaving home in the case of an emergency.	1,036 (79.1)	274 (20.9)
I heat the bread in order to kill the pathogens.	1,043 (79.6)	267 (20.4)
I regularly sanitize highly-touched surfaces at home.	986 (75.3)	324 (24.7)
I bought some facemasks for personal use.	953 (72.7)	357 (27.3)
I do not bring my cellphone out from my pocket to reduce the probability of infection.	870 (66.4)	440 (33.6)
I have made some homemade facemasks for family usage.	210 (16.0)	1,100 (84.0)
I bought some unprescribed drugs to prevent the chance of acquiring the disease.	211 (16.1)	1,099 (83.9)
I allow my children to visit their grandparents'.	116 (8.9)	1,194 (91.1)
I went in-person shopping on days before Nowruz.	92 (7.0)	1,218 (93.0)
I used alcoholic beverages for disinfection.	12 (0.9)	1,298 (99.1)
I attended a memorial of the funeral ceremony of a beloved relative.	13 (1.0)	1,297 (99.0)
On the last Thursday before Nowruz, I went to the graveyard.	14 (1.1)	1,296 (98.9)
I met my family and friends during Nowruz.	8 (0.6)	1,302 (99.4)
I allow my children to have fun with other children outside the home.	7 (0.5)	1,303 (99.5)
I will pay a visit to my family and friends during Nowruz.	5 (0.4)	1,305 (99.6)

## Discussion

Herein, we provided a national survey on the knowledge, attitude, and practice of HCWs during the early days of the COVID-19 epidemic in Iran. HCWs' knowledge score about COVID-19 was satisfactory. More than 90% of HCWs correctly identified the cause of COVID-19, its route of transmission, and major preventive practices. The three most renowned symptoms, including fever, dyspnea, and cough, were correctly recognized by HCWs, which is similar to some other studies (10–12). However, other less-discussed symptoms such as myalgia, malaise, ageusia, anosmia, and gastrointestinal symptoms were less known among HCWs. It could be suggested that this could be due to the fact that the study was conducted in the early months of the epidemic in Iran when many aspects of the disease were still unknown (13, 14). Although more than 90% considered respiratory diseases and age>60 as predisposing factors, almost 65% did recognize hypertension, despite imposing significant hazard, and only 40% realized severe obesity as a prognostic factor. In another study, most HCWs reported that patients with COVID-19 and obesity are more likely to develop more severe conditions (15). While patients with diabetes indicate higher mortality due to COVID-19 compared with patients without any underlying comorbidities (16), 13% of HCWs did not recognize diabetes as a prognostic factor. HCWs' median estimation for the case fatality rate of COVID-19 was 5%, which was similar to the observed case fatality rate of Iran (17). Unlike commonly believed by the public, 92% of HCWs said COVID-19 would also affect the children, which was similar to some other studies (11, 18).

Even though the World Health Organization created a particular webpage to tackle the main misbeliefs and myths regarding COVID-19 infection from the initial days of the epidemic, HCWs had some misbeliefs about COVID-19, too (19). Some 9% of HCWs considered eating garlic to have a preventive effect against COVID-19. Although it has been shown that the transmission of SARS-COV-2 through insects is impossible due to their failure of replicating in mosquitos, as many as 9.5% of HCWs said that COVID-19 could be transmitted via insects (20). Surprisingly, some 16% said that washing hands just with water is enough for sanitization. About 45% said that nasal wash with a saline solution would prevent people from COVID-19. Misbeliefs regarding COVID-19 infection among HCWs were not limited to Iran. Some 16.1% of Nigerian Healthcare workers declared eating garlic can cure COVID-19 (21). Almost 64.4% and 80% of Egyptian HCWs thought COVID-19 is transmissible through arthropods and identified nasal saline washing as a major route of prevention, respectively (22).

Considering the primary source of information about COVID-19 infection, social media, mainly Telegram followed by audiovisual media including radio and television, were significant sources of information among participants. Although social media are promising routes of rapid delivery of health information, it could also be a threat due to disseminating wrong information to undermine the public health response, which is called “COVID-19 infodemics” by the World Health Organization (23). It is worth mentioning that participants who gained information from more scientific platforms, including scientific articles, have better knowledge scores, which underlines the importance or validity of the information sources. Consistently, in this study, we showed that the mean knowledge score of HCWs with professional doctorate degrees was significantly higher than in other groups.

Only 32% of HCWs considered their level of knowledge about COVID-19 to be sufficient. Moreover, 9.0% considered it to be insufficient. Considering the significant role of HCWs in delivering health information to society, it is crucial that they have a sufficient level of knowledge about COVID-19, which is a rapidly evolving health crisis. It is worth mentioning that HCWs who considered their knowledge level about COVID-19 to be sufficient had higher general knowledge scores.

The attitudes of HCWs toward COVID-19 were mostly positive. Some 89% reported that the COVID-19 pandemic would eventually be controlled. Nevertheless, only 64.9% and 71% of Pakistani and Ugandan HCWs believed that the COVID-19 epidemic would finally be contained, respectively (10, 24). Some 88.7% of HCWs correctly recognized the danger of the current situation and the necessity of keeping safe physical distancing, self-isolation upon symptom occurrence, and city lockdowns, which was similar to Pakistan and Egypt (22, 25). Most participants declared that they feel responsible for wearing masks and other preventive measures.

Some 93% of HCWs considered self-isolation and resting at home to be the right measure for COVID-19 patients. Almost all HCWs would notify their employers as soon as the appearance of COVID-19 symptom and HCWs said that they would allow their employees to leave their work, as soon as their COVID-19 symptoms appear. However, it is worth mentioning that workplace-related violence against HCWs would negatively affect this opinion in practice. In Iran, medical education is integrated with hands-on health services. The basis of medical education in governmental hospitals is fundamentally hierarchical and arbitrary and is comparable to that of a military campus (26). In these circumstances, the principal executive forces at hospitals, particularly medical students, interns, and residents, hit the bottom of the authority pyramid and were vulnerable to abuse and violence. In addition, violence is often exacerbated by emergencies, including the current COVID-19 pandemic (27). Therefore, it is to be expected that contrary to what has been stated about being allowed to leave the workplace upon COVID-19 symptoms emergence, the majority of authorities in hospitals would not agree with the HCWs and medical students' sick leave.

Only 14% said that having the pandemic under control would take more than a year. This would suggest the incapability of HCWs to see the bigger picture and their unfamiliarity with pandemics. Thus, it could be suggested that more attention be given to public health at medical schools (28).

HCWs' practice score was sufficient. Some 98% of HCWs said they wore surgical or N95 masks to prevent COVID-19. Previous studies have revealed that 78.8% of HCWs had appropriate practice toward the adherence to COVID-19-related infection control principles (29). The median time of wearing each disposable facemask to reduce the transmission of germs was reported to be 3 h. The median time of washing hands with soap and water was 20 s, which is consistent with the recommendations of the Centers for Disease Control and Prevention (30). Almost all HCWs said they had not traveled nor met their beloved family members during Nowruz. They also declared that they had not visited a graveyard on the last Thursday before Nowruz. The initial 13-days of the Persian new year is called Nowruz, which coincided with the 1st days of COVID-19 epidemic and subsequently social-distancing policy implementations in Iran.

There were major non-scientific practices among HCWs. Almost 10% said that they did not follow the handwashing protocols before touching their face, which was similar to Pakistani HCWs (25). Almost half of HCWs said they took vitamin supplements to prevent COVID-19. A possible explanation is that HCWs are actively involved in counseling the patients with various diseases to consume vitamin supplements, which leads to positivity in their attitude regarding the consumption of vitamins and dietary supplements in general (31). Previous studies have shown higher HCWs' personal use of dietary supplements (32–34). In addition, former reports about the potential impact of dietary supplements toward COVID-19 prevention would augment the tendency of HCWs to consume vitamins (35). Surprisingly of note that there were some HCWs that were involved in quackery. Some 13 participants said they took oral Imam-Kazem-drug for prevention, and three declared the anal usage of violet oil in order to prevent COVID-19. Although investigating regarding complementary and alternative medicine has been of paramount importance since the 1st days of the pandemic, exploiting all unapproved medications is under question due to possible miserable consequences (36, 37). However, it could be suggested that the panic and fear which was bred at the initial days of the epidemic in Iran could lead to current malpractices.

## Strengths and Limitations

The current study is a large nationwide study that aimed to assess the knowledge, attitude, and practice of healthcare workers at the initial days of the COVID-19 epidemic in Iran. The study gathered data from various groups of healthcare workers, including physicians, dentists, pharmacists, nurses, and midwives. The study findings could help authorities to identify major gaps in HCPs' knowledge and practices.

We also acknowledge the limitations of the study. Giving the social-distancing rules during the study period, data were gathered through online platforms. Therefore, the connection to HCWs, who are working in rural areas, was with difficulties. Participants were asked to send the questionnaire to their coworkers in rural areas to ensure higher participation of those groups; however, such voluntary measures are not guaranteed. In addition, as the participants were not asked whether they worked in the public or private sector, the study did not have the power to compare subgroups in this regard.

## Conclusion

The knowledge and practice of HCWs were mostly appropriate and the general attitudes of participants were mainly positive. However, there has still been room for improvement regarding misinformation and quackeries about COVID-19. It could be suggested that educational tools would be the most appropriate way of correcting misconceptions and malpractices.

## Data Availability Statement

The raw data supporting the conclusions of this article will be made available by the authors, without undue reservation.

## Ethics Statement

The studies involving human participants were reviewed and approved by the Ethical Committee of Shahid Beheshti University of Medical Sciences under code IR.SBMU.RETECH.REC.1399.1258. The patients/participants provided their written informed consent to participate in this study.

## Author Contributions

A-AK, HH, S-HG, and MA-K: conceptualization. A-AK, MA-K, and S-HG: data collection and writing—review and editing. MA-K and S-HG: data analysis. HH, S-HG, and MA-K: writing—original draft. A-AK: resources and supervision. All authors have read and approved the manuscript prior to submission.

## Funding

This study was supported by Social Determinants of Health Research Center, Shahid Beheshti University of Medical Sciences under code 25602.

## Conflict of Interest

The authors declare that the research was conducted in the absence of any commercial or financial relationships that could be construed as a potential conflict of interest.

## Publisher's Note

All claims expressed in this article are solely those of the authors and do not necessarily represent those of their affiliated organizations, or those of the publisher, the editors and the reviewers. Any product that may be evaluated in this article, or claim that may be made by its manufacturer, is not guaranteed or endorsed by the publisher.
